# Rho GTPases are Involved on Regulation of Cytodifferentiation of SCC-4 Oral Squamous Cell Carcinoma Cell Line: A Preliminary Study

**DOI:** 10.31557/APJCP.2020.21.1.3

**Published:** 2020

**Authors:** Nanci M Pinheiro, Anna Cecilia D M Carneiro, Virginia O Crema

**Affiliations:** *Department of Structural Biology, Institute of Natural and Biological Sciences, Federal University of Triângulo Mineiro, Uberaba, Brazil.*

**Keywords:** Cytodifferentiation, oral squamous cell carcinoma, Rho GTPases

## Abstract

**Objective:**

This study evaluated the involvement of Rho GTPases proteins in the regulation of cytodifferentiation of the SCC-4 human oral squamous cell carcinoma cell line.

**Methods:**

Cytokeratin and vimentin immunofluorescence and F-actin staining, assays were performed with control cells and *Clostridium difficile *1, 2 and 4 μg/mL Toxin A (Rho GTPases inhibitor) treated SCC-4 cells on three-dimensional Matrigel^TM^ for 24 h. Samples were analyzed by using confocal laser microscopy. Significances were p<0.05.

**Results:**

In all concentrations studied, Toxin A treatment decreased percentage of cytokeratin positive cells (p<0.0001), otherwise, it increased percentage of vimentin positive cells (p<0.0001). There was a negative correlation between cytokeratin and vimentin immunoexpression (p<0.0001), the higher the amount of cytokeratin, the lower the amount of vimentin. Also F-actin polymerization is affected by Toxin A treatment.

**Conclusion:**

Although this is a preliminary study, our results suggest that Rho GTPases are involved on regulation of cytodifferentiation of the SCC-4 human oral squamous cell carcinoma cell line.

## Introduction

Patients with oral squamous cell carcinoma (OSCC) have a high mortality rate (Fazeli et al., 2011) have a poor survival, the 5-years survival rate of approximately 55% to 60% (Rhodus et al., 2014). During carcinogenesis, tumor cells undergo some modifications, such as loss of cytodifferentiation (Kalluri and Weinberg, 2009). Depending on chemical and physical properties of surrounding environment, the GTPases Rho proteins play several inter-connected functions (Sadok and Marshall, 2014).

Rho GTPases are small proteins that regulate complex biological processes such as actin cytoskeleton organization, motility, cell division among others. The Rho subfamily is made up of approximately 23 proteins and, Rho, Rac and Cdc42 groups are the best characterized (Ridley and Hall, 1992). The Rho GTPases are overexpressed in several human tumors (Svensmark and Brakebusch, 2019). The isoforms RhoA, RhoB and RhoC constitute the Rho-like group whose RhoA and RhoC promoting tumor growth while RhoB is a tumor suppressor (Jaffe, 2009). The Rac group, being Rac1 the most important isoform and, Cdc42, participate in the protrusion of lamellipodia and filopodia respectively contributing to tumor cell invasion and metastasis (Svensmark and Brakebusch, 2019).

In cell lines of squamous cell carcinoma of the head and neck the overexpression of RhoA, Rac2 and Cdc42 GTPases in relation to normal keratinocytes has already been demonstrated (Bhave et al., 2011). This study aimed to evaluate the involvement of Rho GTPases on regulation of cytodifferentiation of OSCC cell line by using the *Clostridium difficile* Toxin A treatment, that glucosylates Rho proteins, inactivating all members of Rho GTPases family.

## Materials and Methods

SCC-4 cell line of human oral squamous cell carcinoma of the American Type Culture Collection (ATCC^®^) obtained from the cell bank of the Federal University of Rio de Janeiro was used. Cells were grown in DMEM (Sigma-Aldrich, St. Louis, MO, USA) 1:1 HAM F12 (Invitrogen, Grand Island, NY, USA), 10% FBS (Cultilab Campinas, SP, Brasil), 400 ng/mL hydrocortisone (Ariston, São Paulo, Brazil), 100 U/mL penicillin and 100 μg/mL streptomycin (Sigma-Aldrich, St. Louis, MO, USA) at 37^o^C/ 5% CO_2_. After defrosting of immortalized cells, cells were used in the initial passages. The experiments were performed when the cells reached at least 80% confluence. Biological triplicates and experimental duplicates were performed.

*Immunofluorescence*

1x10^5^ SCC-4 cells/well (biological triplicates) control and *Clostridium difficile* Toxin A - ToxA (List Biological Labs, Campbell, CA, USA) treated: 1, 2 and 4 μg/mL cultured on three-dimensional MatrigelTM for 24 h. 4% paraformaldehyde fixed for 1 h; incubated with: 0.2% Triton X-100 for 5 min, 3% BSA for 20 min, mouse anti-vimentin or anti-cytokeratin clone AE1/AE3 (Santa Cruz Biotechnology, Santa Cruz, CA, USA) at 1:50 overnight , Alexa-488-labelled secondary antibody chicken anti-mouse (Molecular Probes, Eugene, OR, USA) at 1:1,000 for 2 h, rhodamine-conjugated phalloidin (Molecular Probes, Eugene, Ore, USA) at 1:100 for 30 min and DAPI at 1:500 for 15 min. 

Ten immunofluorescence images were obtained randomly at 40x with a laser scanning confocal microscope (Zeiss^®^, Göttingen, Lower Saxony, Germany)) in order to quantify intermediate filaments that are used as a marker of epithelial cells and a marker of mesenchymal cells, cytokeratin and vimentin, respectively. Morphometry was performed by using Zen Blue software (Zeiss^®^). Nuclei DAPI stained were counted and were consider as 100%. The cells stained for cytokeratin or vimentin were counted. The percentage of cytokeratin and vimentin positive cells was calculated by using Microsoft Excel^®^ (Redmond, Washington, Estados Unidos).

*Statistical Analysis*

Levene’s test, Kruskal-Wallis and Dunnet C post-test (vimentin), ANOVA and Tukey post-test (cytokeratin), also Correlation of Pearson were performed by using SPSS 16.0^®^(San Francisco, CA, USA) software and graphics with GraphPad Prism*®* (San Diego, CA, USA). Significance was p<0.05.

**Figure 1:**
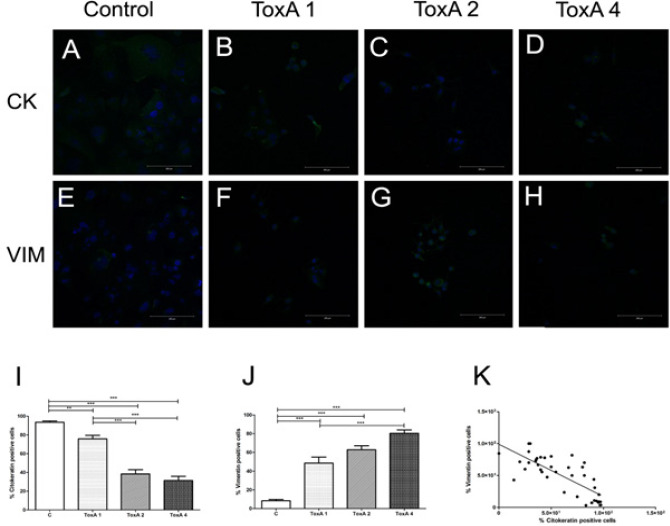
Rho GTPases are Important to Cytodifferentiation of Oral Squamous Cell Carcinoma Cell Line. Confocal analysis from a single section. Nuclei stained with DAPI (blue). Effects of inhibition of Rho GTPases with a broad spectrum inhibitor, *Clostridium difficile* Toxin A on SCC-4 cells: cytokeratin stain (green) control SCC-4 cells (a) and treated cells at 1μg/mL (b), 2μg/mL (c) and 4μg/mL (d); vimentin stain (green): control SCC-4 cells (e), treated cells at 1μg/mL (f), 2μg/mL (g) and 4μg/mL(h). Morphometry of percentual of positive cells for citokeratin (i) and vimentin (j), **p<0.001 and ***p<0.0001. Negative correlation of citokeratin and vimentin immunoexpression, p<0.0001 (k).

**Figure 2:**
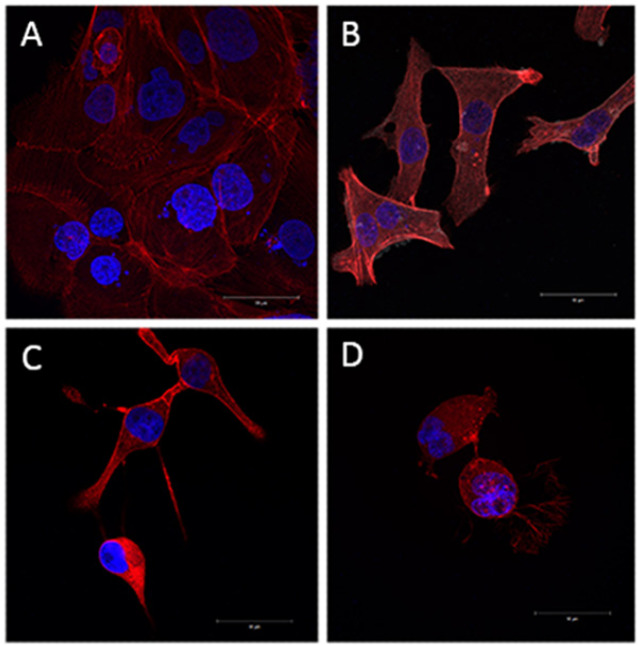
Rho GTPases are Important on Actin Cytoskeleton Organization of Oral Squamous Cell Carcinoma Cell Line. Sequential confocal images were compacted to show the threedimensional aspect. F-actin rhodamine-phalloidin stained (red) and nuclei DAPI stained (blue). Effects of inhibition of Rho GTPases *Clostridium difficile* Toxin A on SCC-4 cells actin cytoskeleton: control cells (a) and treated cells: 1μg/mL (b), 2μg/mL (c) and 4μg/mL (d)

## Results

*Rho GTPases are involved on regulation of cytodifferentiation of the SCC-4 human oral squamous cell carcinoma cell line*

In all concentrations studied, ToxA treatment decreased percentage of cytokeratin positive SCC-4 cells [F(3,39)=62.648, p<0.0001]: 75.8±11.8% (ToxA 1μg/mL), 38.8±14.4% (ToxA 2μg/mL), 31.4±14.0% (ToxA 4μg/mL) compared to 93.5% ±4.5% of control cells (Figure 1a-d,i). Otherwise, in all concentrations studied, ToxA treatment increased percentage of vimentin positive SCC-4 cells [χ(3)=29.010, p<0.0001]: 48.5±20.6% (ToxA 1μg/mL), 62.82±13.39% (ToxA 2μg/mL) and 80.41±11.5% (ToxA 4μg/mL) compared to 8.3±4.9% control cells (Figure 1e-g,j). There was a negative correlation between cytokeratin and vimentin immunoexpression [r=-0.77, n=40, p<0.0001], the higher the amount of cytokeratin, the lower the amount of vimentin (Figure 1k).

As expected, control cells cultured in three-dimensional culture for 24 h showed a well-developed cytoplasm with a prominent cytoskeleton and evident cortex. Cells treated with ToxA (1, 2 and 4μg/mL) showed evident morphological changes when compared to control cells, treated cells presented a reduced cytoplasm, also F-actin and cortical actin polymerization was affected (Figure 2a-d). 

## Discussion

The results found in this study demonstrated the involvement of Rho GTPases proteins in the regulation of cytodifferentiation in oral squamous cell carcinoma cells. Epithelial cells express as marker cytokeratin and mesenchymal cells express vimentin, and the inhibition of Rho GTPases results in stimulation of cytodifferentiation characterized by increased expression of vimentin, an undifferentiated cells marker (Kalluri and Weinberg, 2009). In this study, the cytodifferentiation of SCC-4 cells was affected, decreasing cytokeratin and increasing vimentin immunoexpression, and results showed that the higher the amount of vimentin, the lower the amount of cytokeratin after the Rho GTPases inhibition for 24 hours. Therefore, an inhibition of Rho GTPases results in stimulation of cytodifferentiation characterized by increased expression of vimentin, an undifferentiated cells marker. In this study, the importance of Rho GTPases was demonstrated by their inhibition with *Clostridium difficile* Toxin A (broad spectrum inhibitor of the Rho GTPases family) treatment. *Clostridium difficile* Toxin A exerts an inhibitory effect on all Rho GTPases, and each of them plays a specific role (Zheng et al., 2006).

Head and neck SCC cell lines have high levels of constitutive Rac1 activated that are important to regulate cell invasion, however levels of GTP-RhoA and GTP-Cdc42 were restricted (Patel et al., 2007) and RhoC expression was increased then normal oral epithelium (Kleer et al., 2006). RhoC expression were reduced in tongue SCC cells transfected with ectopic miR-138, and led cells to and changed morphology and increased cell migration and invasion (Jiang et al., 2010). 

The work with immortalized culture makes it possible to manipulate signaling pathways by applying drugs under controlled conditions to analyze biological processes involved with tumorigenesis. On the other hand, it is not possible to understand the interference of various stimuli received by the tumor cell in its microenvironment *in vivo*. Although this study had not used techniques to detect which member of the family of Rho GTPases regulated the biological processes studied, results demonstrated that Rho GTPases are involved on regulation of cytodifferentiation of OSCC cell line. Future studies may elucidate the effect of chemotherapy on specific small GTPases proteins on cytodifferentiation of OSCC cell line.
